# Self-Assembly of Nanocellulose Hydrogels Mimicking Bacterial Cellulose for Wound Dressing Applications

**DOI:** 10.1021/acs.biomac.3c00152

**Published:** 2023-04-25

**Authors:** Linn Berglund, Paula Squinca, Yağmur Baş, Elisa Zattarin, Daniel Aili, Jonathan Rakar, Johan Junker, Annika Starkenberg, Mattia Diamanti, Petter Sivlér, Mårten Skog, Kristiina Oksman

**Affiliations:** † Department of Engineering Sciences and Mathematics, Luleå University of Technology, SE 97187 Luleå, Sweden; ‡ Embrapa Instrumentation, Rua XV de Novembro 1452, 13561-206 São Carlos, São Paulo, Brazil; § Department of Physics, Chemistry and Biology (IFM), Linköping University, Linköping, SE-581 83 Linköping, Sweden; ∥ Center for Disaster Medicine and Traumatology, Department of Biomedical and Clinical Sciences, Linköping University, SE-581 85 Linköping, Sweden; ⊥ S_2_Medical AB, SE-58273 Linköping, Sweden; # Mechanical & Industrial Engineering, University of Toronto, 5 King’s College Road, ON M5S 3G8 Toronto, Canada; ¶ Wallenberg Wood Science Center (WWSC), Luleå University of Technology, SE 97187 Luleå, Sweden

## Abstract

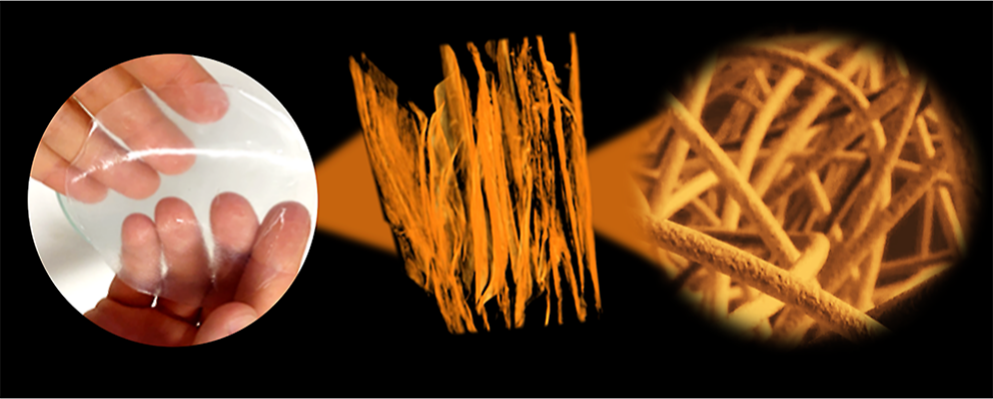

The self-assembly of nanocellulose in the form of cellulose nanofibers (CNFs) can be accomplished via hydrogen-bonding assistance into completely bio-based hydrogels. This study aimed to use the intrinsic properties of CNFs, such as their ability to form strong networks and high absorption capacity and exploit them in the sustainable development of effective wound dressing materials. First, TEMPO-oxidized CNFs were separated directly from wood (W-CNFs) and compared with CNFs separated from wood pulp (P-CNFs). Second, two approaches were evaluated for hydrogel self-assembly from W-CNFs, where water was removed from the suspensions via evaporation through suspension casting (SC) or vacuum-assisted filtration (VF). Third, the W-CNF-VF hydrogel was compared to commercial bacterial cellulose (BC). The study demonstrates that the self-assembly via VF of nanocellulose hydrogels from wood was the most promising material as wound dressing and displayed comparable properties to that of BC and strength to that of soft tissue.

## Introduction

Chronic or non-healing wounds continue to be a public health issue because their prevalence is rising due to population aging, increased longevity, and diseases, such as arterial insufficiency, venous insufficiency, and diabetes.^1^ The field of nanotechnology can provide new approaches to regenerative medicine.^2^ Research on the use of nanocellulose and cellulose in the form of nanosized structures, including cellulose nanocrystals and cellulose nanofibrils, has emerged in the development of engineering strategies for assembly into biocompatible wound dressings.^3−5^

Conventional wound dressing materials can cause wound dehydration and mechanically attach to the wound surface. Thus, replacing wound dressings is an uncomfortable and painful process. In addition to causing persistent pain, distress, anxiety, and chronic morbidity, non-healing wounds result in prolonged hospital stays or even death. For instance, non-healing foot ulcers require amputation in approximately 12% of cases, and the 5 year survival following the procedure is approximately 50%.^6^ Furthermore, the costs of treating chronic wounds are substantial, accounting for approximately 1–3% of the total health care costs in developed countries.^7^ To support and accelerate healing, an ideal wound dressing should provide a layer of protection against external pathogens and further traumas, combined with being biocompatible, so as to not affect the viability of the cells, non-allergenic, comfortable, cost-efficient, and mechanically stable.^8,9^ It should keep the wound moist and also adsorb wound exudates, as the appropriate moisture/exudate balance is essential for the healing mainly of non-healing and chronic wounds.^10,11^

Owing to its resemblance to natural soft tissues such as skin, bacterial cellulose (BC) is the most widely studied type of nanocellulose for skin layer reconstruction.^12,13^ The efficacy of wound dressings made of BC has been demonstrated to decrease the healing time when applied on chronic and acute wounds.^14^ BC is considered a hydrogel, meaning that it is capable of absorbing and holding large amounts of water in a reversible manner while maintaining its structural integrity owing to its three-dimensional hydrophilic network.^15^ It is formed by a fine network of nanofibrils, which can mimic the fibrillar structure of the natural extracellular matrix. Another advantage of bacterial nanocellulose is its transparency, which allows the real-time clinical monitoring of the healing process, which further facilitates the diagnostics of the infection and inflammation in chronic wounds without frequent dressing changes.^16^ However, the large-scale production of BC is limited by the low productivity of known strains, expensive culture media, and high fermentation cost.^17,18^ Moreover, the downstream processing cost of BC for medical applications might be even more expensive to reach the required quality compared to other uses.^19^

Similar to bacterial nanocellulose, plant-derived cellulose nanofibers (CNFs) also possess desirable characteristics for wound dressing applications, such as biocompatibility, high capability to absorb liquid, and simplicity for chemical modifications.^20,21^ CNFs can be produced from numerous renewable and available cellulosic resources.^22−24^ Different chemical pretreatments can be applied to obtain CNFs with specific characteristics,^25,26^ and the mechanical separation technologies available are readily up-scalable.^27^

TEMPO-mediated (2,2,6,6-tetramethylpiperidinyl-1-oxyl-mediated) oxidation is a common chemical pre-treatment generally applied to wood pulp to facilitate the separation and thus the production of CNFs, with characteristics such as uniform widths (∼3 nm) and high aspect ratios (higher than 150), which can provide transparent and strong network formation with increased mechanical performance. TEMPO-CNFs have been applied in a series of studies focusing on biomedical applications in which their biocompatibility has been demonstrated,^28−31^ as well as their antibacterial properties after autoclaving.^32^

The up-scalable preparation of high-performance functional hydrogels that are able to mimic native tissue microenvironments, combined with adequate mechanical performance in a swollen state, remains one of the challenges for the effective transference of hydrogels for practical uses.^33,34^ For wound dressing applications, CNFs are often utilized as reinforcement in multicomponent composite hydrogels^35^ where they are combined with other components such as nanostructured elastomers^36^ to mimic the mechanical properties of human skin (2.9 to 150.0 MPa).^37,38^ To improve biocompatibility and swelling capacity, combinations of collagen, chitosan, and alginate have also been reported,^39^ aiming to achieve the required multifunctionality of wound dressings. Different assembly strategies of hydrogels, such as alternating layered structures^40^ as well as double-network^12,41^ and triple-network^42^ structures have been evaluated to enhance their mechanical performance.

Despite recent progress in designing multifunctional hydrogels with promising mechanical properties, the preparation strategies are complex and involve several processing steps that are difficult to scale up and add to the risk of introducing components that can exhibit cytotoxic side effects, which is problematic for applications such as wound dressing.^43^

The significance of utilizing the intrinsic strong network-formation ability of CNFs in the assembly of hydrogels with favorable mechanical properties has not been fully recognized. Although templating strategies for nanofiber self-assembly via suspension casting and vacuum filtration are very common methods that have been extensively applied to assemble dried nanofiber networks/films, or so-called nanopapers^22,26,40,44−49^ for various applications, these techniques have not been evaluated for the self-assembly of CNF-based hydrogels alone, without the addition of any other component or crosslinking agents.

The objective of this study was to allow CNFs to self-assemble via hydrogen-bonding assistance into up-scalable, highly efficient, versatile, and completely bio-based hydrogels. First, negatively charged TEMPO-oxidized CNFs were separated directly from wood (W-CNFs) and compared with CNFs separated from wood pulp (P-CNFs). Second, two approaches were evaluated for hydrogel assembly. Water was removed from the suspensions via evaporation through suspension casting, allowing the self-assembly of inter-fibril bonds into a strong network, or via vacuum-assisted filtration. Finally, the hydrogels were compared to BC according to their application potential as wound dressings.

## Experimental Section

### Raw Materials and Chemicals

Hardwood aspen powder (300–500 μm) was compared with never-dried dissolving softwood pulp provided by Domsjö Fabriker (Örnsköldsvik, Sweden) as the starting material for the preparation of CNFs. The aspen wood had cellulose, hemicellulose, and lignin contents of 45–52, 20–28, and 20–24%, respectively.^50^ The softwood pulp had cellulose, hemicellulose, and lignin contents of approximately 95, 4.5, and 0.1%, respectively.^51^ High-purity sodium chlorite (77.5–82.5%), standard hydrochloric acid solution (0.5 N), standard sodium hydroxide solution (0.1 N), and sodium hydroxide beads (≥97%, ACS) were purchased from VWR International LLC (Stockholm, Sweden). 2,2,6,6-Tetramethylpiperidin-1-oxyl (TEMPO) (99%), glacial acetic acid (CH_3_COOH, 100%), sodium hypochlorite (NaClO, 6–14% active chlorine), and sodium bromide (≥99%) were purchased from Sigma-Aldrich AB (Stockholm, Sweden). Lyophilized bovine serum albumin (BSA) and phosphate-buffered saline (PBS) were also purchased from Sigma-Aldrich AB (Stockholm, Sweden) and used at a BSA concentration of 50 g L^–1^ in PBS for swelling capacity characterization. All chemicals were used as received, and distilled water was used for all experiments. BC produced from *Komagataeibacter xylinus* was provided by S2Medical AB (Linköping, Sweden) and used as a reference material.

### Preparation of CNFs

Starting materials were treated by the TEMPO/NaClO/NaClO_2_-system following a method described by Jonasson et al. (2021).^24^ Primary oxidant NaClO_2_ (5.0 mg g^–1^ dry matter) and TEMPO (17.5 mg g^–1^ dry matter) were added together with a phosphate buffer (0.1 M, 100 mL, pH = 6.8) at a liquor: dry matter ratio of 100:1. The flask was sealed and submerged in an oil bath, after which NaClO (1 mL g^–1^ dry matter) was added and kept at a temperature of 60 °C for 72 h. After cooling the suspension to room temperature, the material was washed until neutral pH was achieved. After the TEMPO oxidation, the suspension was diluted to 0.2 wt %, followed by homogenization with a high-pressure homogenizer APV 2000 (SPX Flow Inc, Silkeborg, Denmark) at 1000 bar and collected after 1 pass. The materials are denoted as W-CNF and P-CNF, respectively.

The viscosity of CNFs was measured after fibrillation at a concentration of 0.2 wt % using a Vibro Viscometer (SV-10, A&D Company Limited, Tokyo, Japan) with a tuning fork vibration method at a vibrational frequency of 30 Hz. Viscosity was used to characterize degree of fibrillation when comparing W-CNF and P-CNF, respectively.

The degree of carboxylation of CNFs was quantified by conductometric titration. Approximately, 50 mL of the CNF suspension was diluted in 0.05 M HCl. Titration with 0.01 M NaOH was then conducted in triplicate to determine the amount of weak acid groups (mmol g^–1^) present on the nanofiber surface.

The surface charge was also compared for W-CNF and BC using a Zetasizer Nano Z (Malvern Pan Analytical Ltd, Malvern, UK) at room temperature (RT, 23 °C ± 2), in triplicate. Prior to the measurements, the samples were diluted to 0.05% (w/v), and BC was disintegrated using a high-shear mixer to achieve a measurable suspension.

### Hydrogel Self-Assembly

For all hydrogels, suspensions were diluted to 0.2 wt % in water under magnetic stirring for 1 h and subsequently degassed for 12 h under vacuum to remove air bubbles. For self-assembly via vacuum-assisted filtration, the suspension was poured in a Büchner funnel with filter paper (Whatman grade 52, pore size of 7 μm, 90 mm diameter) and metal wire mesh at RT for 4–24 h depending on the grammage (g of CNFs per m^2^ surface) of the hydrogel, which was between 10 and 120 g m^–2^. After the network was formed and dried, the hydrogel was submerged in water and the filter paper was removed. For self-assembly via the suspension casting technique, the suspension was poured into a Petri dish and left to evaporate at RT until the network was formed after up to 6 days. Thicknesses of the hydrogels in the dry state and in the wet state of equilibrium water absorption were measured using a thickness gauge.

### Optical Microscopy

The microstructure of the starting materials was studied using an optical microscope (Nikon Eclipse LV100N POL, Kanagawa, Japan) and the imaging software NIS-Elements D 4.30.

### Atomic Force Microscopy

W-CNFs and P-CNFs were studied according to their morphology and dimensions using a Veeco MultiMode Scanning Probe atomic force microscope (Bruker, Santa Barbara, CA, USA) in the tapping mode with a tip model TESPA [antimony (*n*) doped silicon] (Bruker, Camarillo, CA, USA). Diluted suspensions (0.01 wt %) were dropped onto a clean mica surface and left to dry at RT. The nanofiber widths were determined from the height images using Nanoscope V software, and the average values and deviations presented here were based on 100 separate measurements.

### Scanning Electron Microscopy

Cross-sections of W-CNF-SC, W-CNF-VF, and BC were examined using scanning electron microscope JEOL JSM 6460LV (JEOL Ltd., Tokyo, Japan) at an acceleration voltage of 15 kV. The samples were prepared by freeze-drying and platinum coating using an EM ACE200 sputtering instrument (Leica, Wetzlar, Germany) before observation to avoid electron charging. A coating layer 15 nm thick was deposited in a vacuum of 6 × 10^–5^ mbar under a current of 100 mA.

### X-ray Microtomography

The structures of W-CNF-SC, W-CNF-VF, and BC were 3D reconstructed using a Zeiss Xradia 510 Versa, Carl Zeiss (Pleasanton, CA, USA) with a 20× objective, performing interior tomography with a field of view of 0.56 mm and voxel size of 0.56 μm. Samples of approximately 2 mm^3^ were scanned, with the region of interest positioned precisely at the center of each sample. Scanning was performed with an X-ray tube voltage of 50 kV, output power of 4 W, and no X-ray filters. A total of 2401 projections with an exposure time of 6 s resulted in a total scan time of 6 h. Reconstruction was performed using filtered back-projection with Zeiss Scout-and-Scan Reconstructor software (version 11.1). 3D visualization and analysis were performed using the Dragonfly Pro Software (ORS).

### Absorption Measurements

CNF and BC hydrogels in the dry state were immersed in water at RT to study their swelling capacity over time. The wet weights of the hydrogels were recorded at regular intervals for 72 h. Excess water was removed by gently tapping the samples on a dry tissue paper. The absorption was calculated as follows
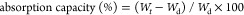
1where *W*_d_ denotes the initial weight of the dried sample and *W*_*t*_ is the weight at time *t* after immersing the samples in water. In addition, the absorption capacity was evaluated in PBS and 50 g L^–1^ BSA in PBS to resemble the wound fluid for the W-CNF-VF hydrogel of 10 g m^–2^ and BC hydrogels.

### Water Retention Measurements

Water vapor transmission rate (WVTR) was measured to evaluate the moisture maintenance of the hydrogels. WVTR was calculated according to SS-EN 13726-2 with minor modifications. W-CNF-VF-20 and BC samples were equilibrated in Milli-Q water for a minimum of 30 min and cut in discs of 12 mm *Ø*. The membranes were positioned on the mouth of small glass vials filled with 3 mL of distilled water and secured with the help of a holed screw cap. The system was sealed with the wax film to exclusively ensure vapor diffusion through the 64 mm^2^ orifice. The test was carried on in 37 °C in 32% RH and the samples were conditioned for 6 h prior to the beginning of the test. The vapor evaporation through the membrane was evaluated through periodic weighting over 4 days. Weight loss data were normalized against the hydrogel area and time, allowing the calculation of the WVTR. Four samples were tested per each condition.

The hydrogels were also evaluated according to their water retention ratio (WRR). Samples were incubated in Milli-Q water for at least 30 min prior to the measurement. Discs were cut with a biopsy punch and blotted with wet tissue paper to remove excess water. The samples were positioned on paraffin wax in an open mouth container and allowed to dry in room temperature. The weight loss was monitored with constant weighting at 30 min intervals. The samples were subsequently dried in a desiccator cabinet to determine dry weight. The WRR was evaluated according to the formula
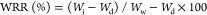
2where *W*_i_ corresponds to the instantaneous weight, *W*_d_ corresponds to the dry weight, and *W*_w_ corresponds to the initial weight of the samples. Measurements were taken in quintuplicates.

### Mechanical Testing

The tensile properties of the CNF and BC hydrogels were measured using a Shimadzu Autograph AG-X universal testing machine (Shimadzu Corp., Kyoto, Japan) equipped with a 1 kN load cell. The tests were performed at a strain rate of 2 mm min^–1^ and a gauge length of 20 mm. The samples were in the form of strips that were 6 mm in width and 80 mm in length and were tested in the hydrogel state (at a dry content of 10.5 wt %). The average values reported were based on 10 measured samples.

A cyclic compression test was carried out at 25 °C using a dynamic mechanical analyzer (DMA Q800, TA Instruments, New Castle, DE, USA). The 120 g m^–2^ W-CNF-VF hydrogel sample was compressed for three compression cycles. The samples were preloaded with a 0.05 N load and compressed up to a strain of 100% at a strain rate of 10% min^–1^. The samples were left in air for a dwell time of 5 min between the compression cycles or were immersed in water for 5 min between the cycles to study the hydrogel recovery.

### Rheology Measurements

The measurements were performed on a Discovery HR-2 rheometer (TA Instruments, New Castle, USA) with a protocol adjusted for material characterized by short relaxation times.^52^ A 8 mm parallel plate geometry was employed, and all measurements were carried out at 25 °C. Temperature regulation was obtained with a Peltier system. 8 mm discs were prepared with the help of a biopsy punch, and the materials were incubated in Milli-Q water for at least 30 min prior to the test. The samples were sequentially compressed and allowed to relax. Compression to axial force levels of 0.1, 0.5, 1, 2, 4, and 6 N were performed with a compression speed of 5 μm s^–1^. Subsequently, the gap size was maintained at constant while the samples were allowed to relax for 5 min. The viscoelastic properties were assessed in this step with small amplitude oscillatory deformations at 1 Hz frequency and 0.01% strain (in the linear viscoelastic region). The samples were run in triplicates.

### Thermogravimetric Analysis

The thermal stability of the W-CNF-VF hydrogel of 10 g m^–2^ and BC hydrogels was evaluated using a thermogravimetric analyzer (TGA) Q500 from TA Instruments, New Castle, DE, USA. The samples were heated under a nitrogen atmosphere in a platinum pan from 30 to 900 °C at a heating rate of 10 °C min^–1^. All samples were dried in a vacuum oven at 50 °C, overnight to remove the moisture.

### Primary Cell Isolation and Culture

Primary dermal fibroblasts were isolated from human tissues obtained from healthy patients undergoing reduction abdominoplasty at the University Hospital in Linköping, Sweden. All human tissues were handled in accordance with the guidelines postulated by Linköping University with approval from the Swedish Ethical Review Authority (no. 2018/97-31). Primary cells were isolated by mechanical dissection and enzymatic digestion of the dermis under sterile conditions. Skin samples were repeatedly washed in sterile PBS and the subcutaneous fat was mechanically removed. The remaining dermis was dissected into 1 × 3 mm^2^ pieces and placed in Dulbecco’s modified Eagle’s medium (DMEM, Gibco Thermo Fisher Scientific, Paisley, U.K.) with 165 U mL^–1^ collagenase (Gibco Thermo Fisher Scientific, Paisley, UK) and 2.5 mg mL^–1^ dispase (Gibco Thermo Fisher Scientific, Paisley, UK) and incubated at 37 °C, 5% CO_2_, and 95% humidity for 12 h. After enzymatic digestion, the suspension was centrifuged for 5 min at 200*g* and the resulting cell pellet was resuspended in the fibroblast medium (DMEM containing 10% fetal calf serum, 50 U mL^–1^ penicillin, and 50 mg mL^–1^ streptomycin). The cells were seeded into 75 cm^2^ culture flasks (Falcon, Corning Inc.; Corning, NY, USA) in a fibroblast medium and kept in an incubator at 37 °C, 5% CO_2_, and 95% humidity. The medium was changed three times per week.

### Cell Proliferation

Following the establishment of primary cultures, the cells were enzymatically detached using 0.02% versene/0.1% trypsin and seeded in 12-well plates (Falcon, Corning Inc., Corning, NY, USA) at a density of 10,000 cells cm^–2^. The cells were allowed to adhere for 24 h and subsequently covered with *Ø* 6 mm discs of W-CNF. Fibroblast cultures without the addition of the material served as controls. Every 24 h for 72 h in total, cells were detached using a 0.02% versene/0.1% trypsin solution at 37 °C for approximately 10 min, stained with trypan blue to distinguish viable cells, and counted using an EVE automated cell counter (NanoEnTek, Waltham, MA, USA). All experiments were performed in biological triplicates (separate cell cultures) and methodological duplicates (staining and cell counting). The number of viable cells was recorded and analyzed using Prism 8.4.2 (GraphPad LLC, La Jolla, CA, USA). A two-way ANOVA coupled with a Holm–Sidak post-test was used to compare cell numbers over time in all groups of the same cell type, and *p* < 0.05 was considered significant. The cell numbers at any given time point were expressed as a proliferative index according to the following equation
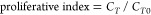
3where *C*_*T*_ denotes the mean number of viable cells at the analyzed time point and *C*_*T*0_ denotes the mean number of viable cells at the starting time point. All values are plotted as mean ± standard deviation unless otherwise stated.

## Results and Discussion

### Nanocellulose from Wood or Pulp—Evaluation Based on Network Formation

**Figure 1:**
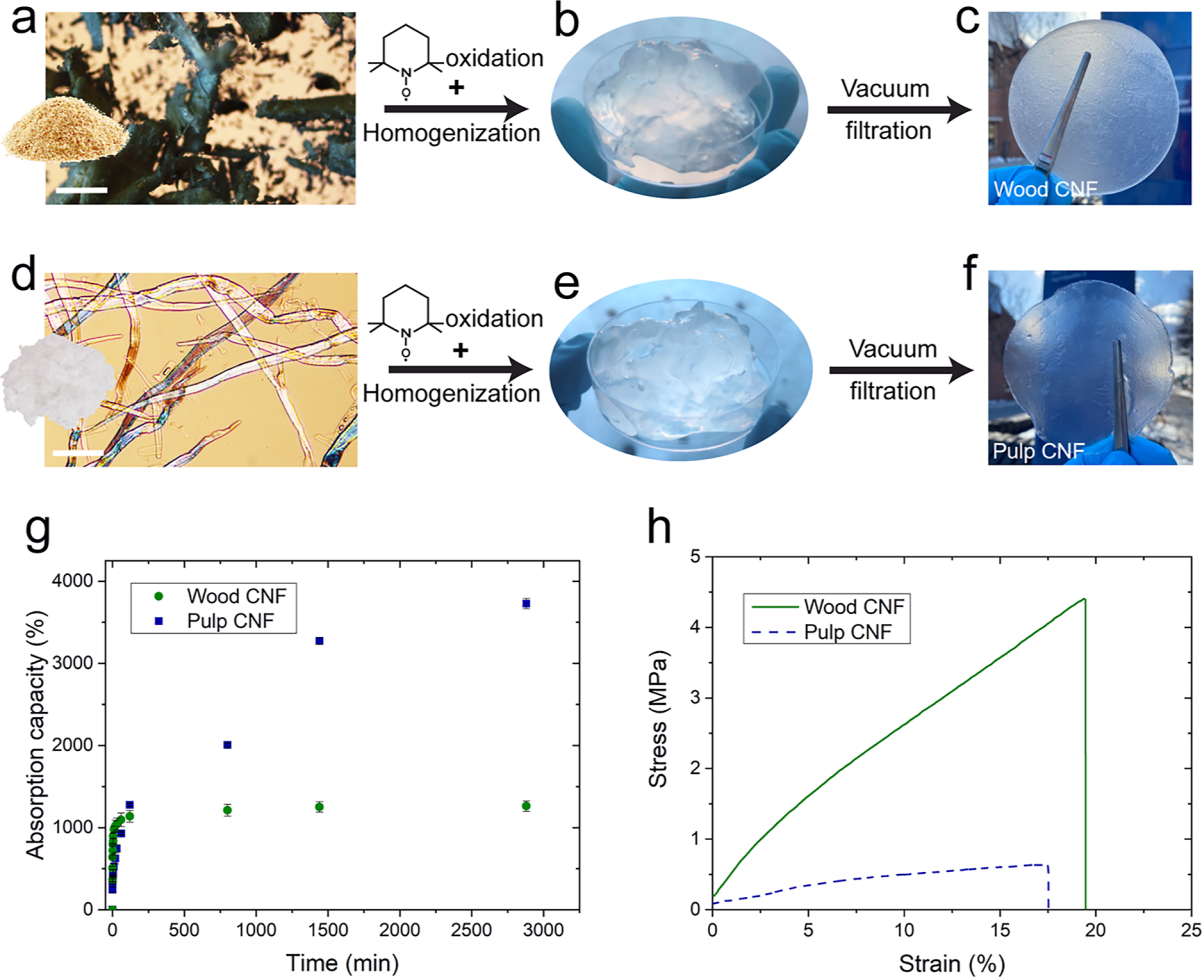
(a–f) Schematic presentation of the processing routes from the two starting materials (a) wood powder and (b) pulp, to self-assembly via vacuum-assisted filtration of CNF suspension into hydrogels. Optical microscopy (OM) images of (a) wood powder scale bar: 500 μm, (d) pulp fibers scale bar: 100 μm, photographs of (b,e) CNF gel (1 wt %), and (c,f) assembled hydrogel (40 g m^–2^) of W-CNF and P-CNF, respectively, after immersion for 48 h in water. (g) Water absorption capacity of the hydrogels (40 g m^–2^). (h) Representative tensile stress–strain curves of the two CNF hydrogels at 40 g m^–2^, tested after 1000% absorption.

W-CNFs and P-CNFs were compared in terms of their network formation ability that is an important characteristic to obtain strong and stable hydrogels. An overview of the raw materials used for CNF hydrogels is presented in Figure 1 along with the water absorption capacity and mechanical properties of the hydrogels.

The morphology was characterized by atomic force microscopy (AFM) and the average nanofiber widths were measured from AFM height images to 1.9 ± 0.9 and 2.1 ± 0.8 nm for W-CNF and P-CNF, respectively (Figure S1, Supporting Information). The hydrogels were allowed to self-assemble under vacuum-assisted filtration from both CNFs (Figure 1a–f) for an initial assessment of their network characteristics and suitability as hydrogels for wound dressing applications. The water content and swelling of hydrogels are two relevant properties, as the wound healing process is facilitated by a moist environment and a suitable swelling capacity is beneficial for the absorption of wound exudates as it reduces the chances of bacterial infection.^53^ A comparison of the swelling kinetics displayed in Figure 1g shows that the P-CNF hydrogel did not reach the equilibrium state and exhibited an uncontrolled absorption behavior; on the contrary, the hydrogel prepared with W-CNF reached the equilibrium condition in a short period of time. The P-CNF hydrogel could be visually distinguished from W-CNF by its less stable network formation and it was difficult to handle without damaging its structural integrity. The higher absorption capacity of the P-CNF hydrogel could be attributed to a more negative surface charge after TEMPO-oxidation compared to that of W-CNF (the degree of carboxylation was 0.45 ± 0.12 mmol g^–1^ for W-CNF and 0.72 ± 0.13 mmol g^–1^ for P-CNF). These results are in agreement with those reported in the literature, showing that a higher content of carboxylate groups results in higher repulsive forces between TEMPO-oxidized nanofibers, which in turn increases the absorption capacity in water.^54,55^

The mechanical properties of the hydrogels are shown in Figure 1h, where the W-CNF formed a stronger network with higher stiffness and strength compared to P-CNF. The combination of high yet controlled swelling and high mechanical properties reflects the superior ability of hydrogels prepared with W-CNFs to form stronger and more stable networks compared with those of P-CNFs. The measured viscosity of the nanofiber gel prior to self-assembly was 26.2 ± 2.2 and 6.7 ± 1.3 mPa s for W-CNFs and P-CNFs, respectively. These results furthermore indicate a stronger network formation ability of W-CNFs compared to P-CNFs. Overall, the structure–property relationship of CNF networks has been extensively researched and shown to be dictated by a variety of factors, such as CNF isolate origin,^22,56^ chemical composition,^26,57^ CNF size,^58^ porosity,^59^ and humidity.^60^ Apart from the different surface charges of the CNFs, the stronger network formation of CNFs from wood could be attributed to the presence of hemicellulose to a higher extent in W-CNF, which has been shown to act as a binder between cellulose nanofibrils,^26,56^ and exert distinct mechanical contributions to CNF networks.^61^

A serious decline in the mechanical properties of polysaccharide hydrogels caused by high or uncontrolled absorption is a difficult problem that greatly limits their application, especially in the medical field. The W-CNF-VF hydrogels displayed controllable swelling combined with stable mechanical properties at the initially prepared grammage of 40 g m^–2^, although the thickness of the hydrogel limited its conformability as well as transparency to some extent (Figure 1c).

In a previous study, the grammage (i.e., amount of CNFs per surface area) of a hydrogel was one approach to adjust hydrogel properties, such as thickness, transparency, absorption capacity, and mechanical properties.^57^ The lower and upper limits of self-assembled W-CNF-VF hydrogels were studied to establish a feasible and practical range according to hydrogel stability and handling, as presented in Table 1.

**Table 1:** Theoretical Grammage, Actual Grammage in Brackets, and Equilibrium Absorption Capacity of Hydrogels Prepared with W-CNFs at Different Grammages via Vacuum-Filtration

sample code	grammage (g m^–2^)	equilibrium absorption capacity (%)
W-CNF-VF-10	10 (6.9 ± 0.7)	3275 ± 190
W-CNF-VF-20	20 (19.0 ± 0.3)	1024 ± 67
W-CNF-VF-30	30 (27.6 ± 0.8)	1122 ± 170
W-CNF-VF-60	60 (58.8 ± 3.5)	2751 ± 174
W-CNF-VF-80	80 (78.6 ± 1.0)	2490 ± 298
W-CNF-VF-120	120 (117.5 ± 3.5)	2600 ± 233

**Figure 2:**
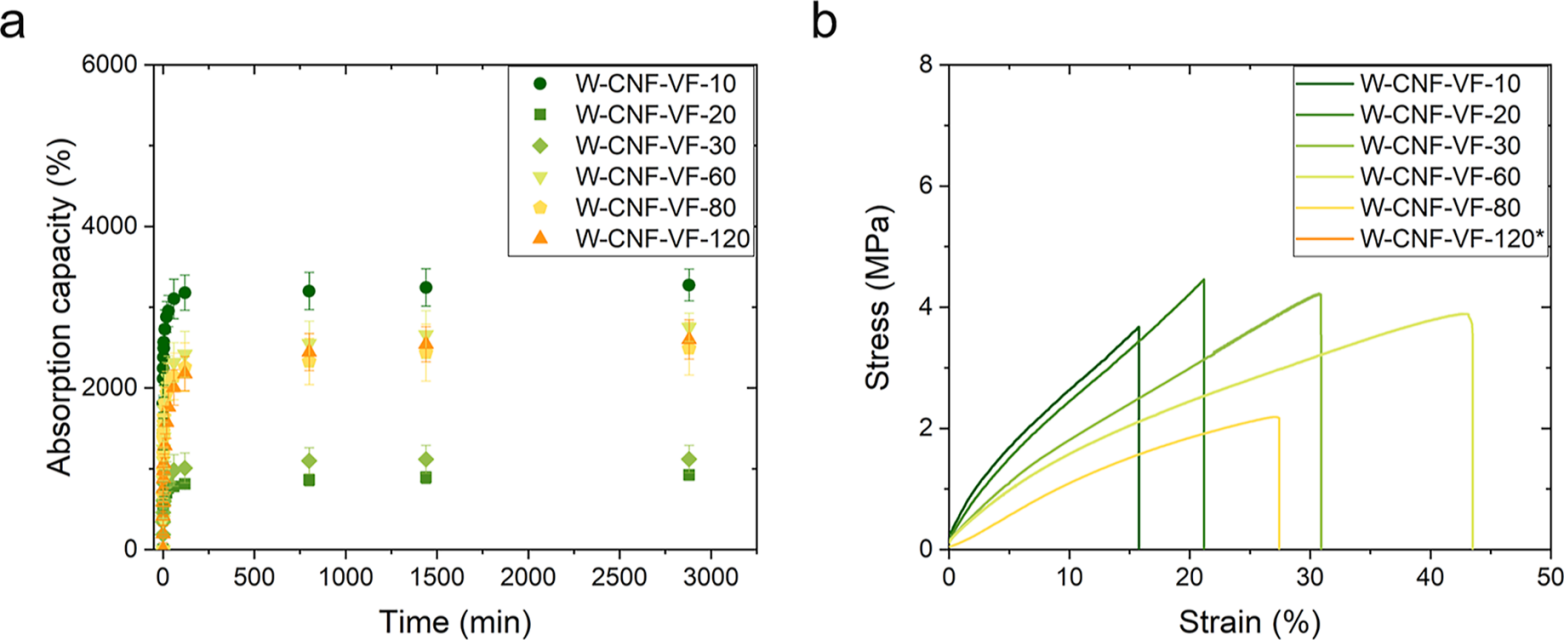
(a) Water absorption capacity of the vacuum-filtered W-CNF hydrogels at different grammages. (b) Representative tensile stress–strain curves of the W-CNF-VF hydrogels at different grammages. *120 g m^–2^ hydrogel could not be tested in tensile.

The effect of grammage on the absorption (Table 1) and mechanical properties was studied for the W-CNF-VF hydrogel at different grammages, as presented in Figure 2.

The initial and theoretically calculated values of hydrogels via vacuum-assisted filtration and the real grammage measured after hydrogel preparation were different (Table 1). The actual grammages were lower for all materials, suggesting that some material was lost in this step and the vacuum-filtration yields were between 70 and 98%. The water absorption capacity (Table 1; Figure 2a) was initially reduced with increasing grammage up to 30 g m^–2^ followed by enhanced absorption when the grammage was further increased to 120 g m^–2^. As a multilayered structure is typically found in nanofiber networks,^57,62−66^ the increased fiber content (grammage) likely provides the formation of more layers. Initially, additional layers, or the formation of a thicker layer, appear to hinder the water uptake up to a certain point, after which the CNFs can be packed in a dense network structure, with very low porosity, which seems to be impaired and contributes to the formation of voids and, in turn, enhances the water uptake.

When subjected to tension, the CNF network exerts an interchain sliding mechanism, and the fracture involves a cascade of hydrogen bond breaking and re-forming.^40^ Regarding the mechanical properties (Figure 2b), increasing the grammage from 10 to 60 g m^–2^ simultaneously had a positive effect on the tensile strength and strain, that is, elongation to break, while the elastic modulus was reduced. For grammage values higher than 60 g m^–2^, the overall mechanical properties decreased. The hydrogel of 120 g m^–2^ could not be clamped in the tensile testing grips without damaging its structural integrity; thus, its tensile properties could not be tested. Similar trends have been observed for CNF networks, whereby the tensile properties increase with increasing grammage in the range of 5–58 g m^–2^.^63,67^ The mechanical network properties rely on hydrogen bonding networking and interfacial interactions, and if the CNFs cannot be effectively packed into dense networks at higher grammage values, the hydrogel structure will be influenced and, in turn, as will its ability to transfer load and absorption capacity.

### Hydrogel Self-Assembly via Suspension Casting and Vacuum-Assisted Filtration Techniques

**Figure 3:**
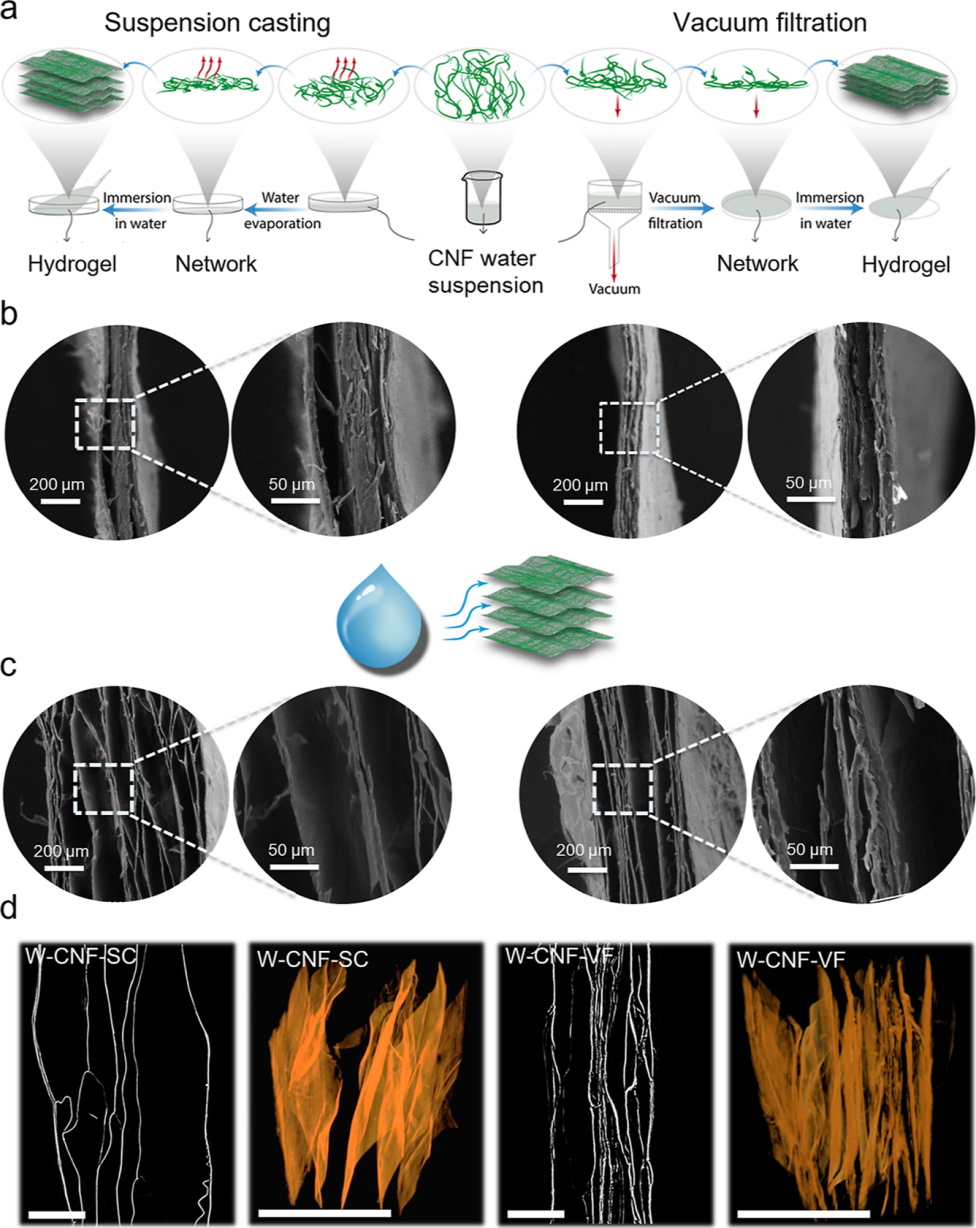
(a) Setup for the CNF hydrogel self-assembly via suspension casting (W-CNF-SC) and vacuum-assisted filtration (W-CNF-VF) techniques. A freeze-dried hydrogel grammage of 120 g m^–2^ visualized using scanning electron microscopy (SEM) (b) before absorption and (c) after absorption to 1000% for W-CNF-SC and W-CNF-VF, respectively. (d) Visualization in 2D and 3D X-ray microtomography (XRT) reconstruction of a part of the cross-sections after 1000% absorption. Scale bar: 100 μm.

**Figure 4:**
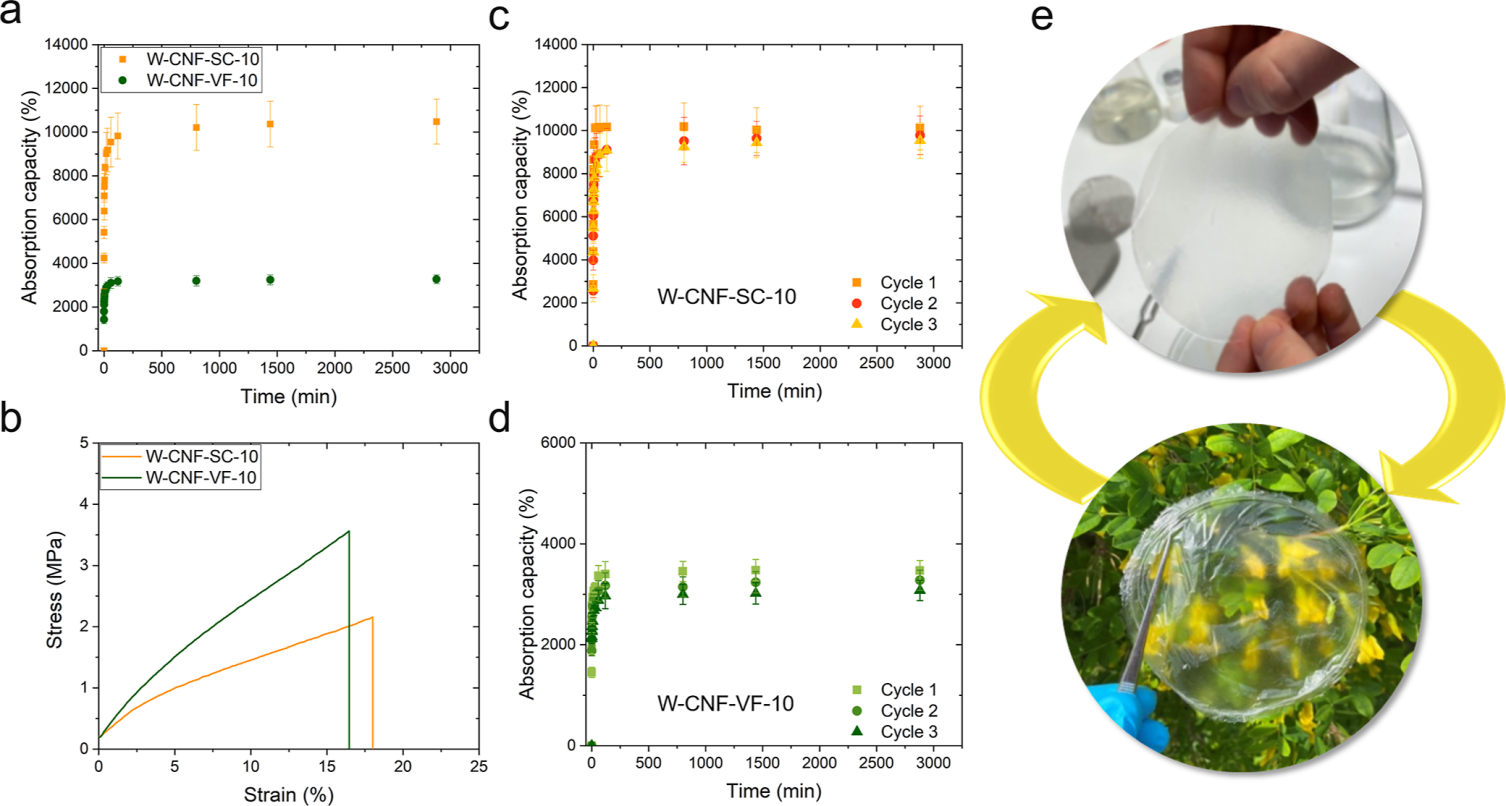
Hydrogels by the SC and VF of grammage (10 g m^–2^). (a) Water absorption capacity and (b) representative stress–strain curves. Absorption capacity after cyclic drying and re-swelling. (c) W-CNF-SC and (d) W-CNF-VF hydrogels and (e) representative photographs in the hydrogel state and dried state.

CNFs from wood were allowed to self-assemble via the evaporation of water through suspension casting (W-CNF-SC) and vacuum-assisted filtering (W-CNF-VF). CNFs are mechanically entangled in the wet gel, and as water evaporates or is filtered, capillary forces provide attraction between individual nanofibers. As they come close enough, secondary attraction forces, including hydrogen bonding, develop between CNFs, and the resulting network structures possess an interesting combination of properties. The differences between hydrogel self-assembly via suspension casting and vacuum-assisted filtration techniques and the properties of the resulting hydrogels are compared and summarized in Figures 3 and 4, respectively.

For microscopic analysis, the highest grammage of 120 g m^–2^ was studied as it had sufficient thickness to show variations in the network before and after absorption (Figure 3b–d). The high-grammage hydrogels were also evaluated by cyclic compression testing, and the representative stress and strain curves are provided in Figure S2 (Supporting Information). A lower grammage value of 10 g m^–2^ was also assembled using the two techniques, and their absorption capacity, mechanical properties, and ability to reswell after cyclic drying were evaluated (Figure 4a–e).

The self-assembly based on both techniques presented in Figure 3a resulted in a layered structure (Figure 3b), where the cross-section was visualized prior to absorption in water. W-CNF-SC appeared to form a less compact layered structure than W-CNF-VF. The equilibrium capacity, i.e., maximum swelling of the hydrogels were achieved at 4150 and 2600% for W-CNF-SC and W-CNF-VF, respectively. To compare the swelling behavior of the hydrogels, they were submerged in water until 1000% absorption capacity was reached and subsequently freeze-dried before scanning electron microscopy (SEM) analysis (Figure 3c) and X-ray microtomography (XRT) reconstruction (Figure 3d). As shown in Figure 3c, upon swelling, water appears to penetrate the structures between the dense layers, resulting in separation between the CNF network layers to enable absorption. By comparing the different structures, it is possible to observe the swelling mechanism in which the layers are more separated with increasing water absorption and that it occurs to a larger extent for the W-CNF-SC hydrogel, which reaches a higher equilibrium absorption capacity compared to the W-CNF-VF hydrogel. This was also observed in the 2D and 3D reconstruction of the layered structure, showing a larger space between the layers of the casted hydrogel. The higher equilibrium absorption capacity of W-CNF-SC could be explained by less interconnectivity between the layers that stabilize the structure, where fewer attached layers can facilitate the accessibility of water to enter the structure (Figure 3c,d).

Based on Figure 4a, the same behavior can be observed for the lower-grammage hydrogels, where the difference in absorption capacity is even more pronounced and at an equilibrium as high as 10,485 ± 1024% for W-CNF-SC, compared to 3275 ± 190% for W-CNF-VF. The nanocellulose hydrogels revealed an impressive ability to dry, for potential long-term storage or shipment, and were reswelled to the hydrogel state and their thicknesses in dry and wet states are presented in Table S1. This characteristic was evaluated over three cycles (Figure 4c–e). Photographs of the W-CNF-SC and W-CNF-VF hydrogels after drying and re-swelling are provided in Figure S3 (Supporting Information). Cyclic drying did not appear to negatively affect the hydrogel absorption capacity, which was maintained at comparable values upon cyclic drying and re-swelling for both the W-CNF-SC and W-CNF-VF hydrogels. This behavior was also observed for a grammage of 20 g m^–2^; the absorption capacity as a function of time is shown in Figure S4 (Supporting Information).

Mechanical properties, such as the tensile strength and elongation to break, are important for wound dressings, as the material should not break during the fixation process, wearing, or removal.^68^ Benítez et al. (2013) reported that as the moisture increases, the elastic modulus, tensile strength, and toughness of cellulose networks gradually decrease, as the absorption of moisture weakens the bonding strength between CNFs owing to the reduction of hydrogen bonds, resulting in easier interfacial debonding and sliding between CNFs.^60^ Still, the lower grammage hydrogels exhibited excellent mechanical performance with a tensile strength of 2.2 ± 0.4 MPa, strain of 18.1% ± 2.4, and an elastic modulus of 12.3 ± 1.3 MPa for W-CNF-SC, compared to the overall higher properties of W-CNF-VF with a tensile strength of 3.9 ± 0.1 MPa, strain of 17.0% ± 1.2, and an elastic modulus of 19.7 ± 0.9 MPa (Figure 4b).

The vacuum filtration technique has previously been shown to result in a mechanically stronger nanofiber network than the casting method.^69^ However the reason for this has not been explained. In our study, the hydrogels were tested at a comparably high absorption of 1000%; however, the difference in structures combined with the effect of water interaction could plausibly explain the difference in mechanical properties. The water molecules that disrupt the resistance to sliding by decreasing the number of hydrogen bonds in the wet interface results in a greater effect on the less interconnected layers of the cast structure, as shown in Figure 3.

In addition, the two techniques were further compared according to the mechanical stability of the W-CNF-SC and W-CNF-VF hydrogels by cyclic compression testing at a grammage of 120 g m^–2^. The highest grammage was chosen for adequate hydrogel thickness to be tested at the equilibrium absorption capacity under compression. However, the W-CNF-SC hydrogel did not exhibit the stability to be handled at this high grammage and swelling and thus could not be tested in compression.

Meanwhile, the cyclic compression data for the W-CNF-VF hydrogel (Figure S2) showed high stability and recovery after compression. To date, few studies have discussed the effects on CNF networks in the hydrogel state. Based on these experimental results, the theoretical structure–property relationship between the absorption and mechanical properties of self-assembled CNF hydrogels should be further studied.

From a practical perspective, both techniques can be applied for large-scale production. However, it should be noted that although casting does not require any energy input, it is a more time-consuming method than vacuum-assisted filtration, hours compared to days for drying, respectively. Altogether, W-CNF-VF displayed excellent water absorption capacity combined with mechanical stability both in tensile and compression tests and was thus chosen as the most promising candidate for further characterization and comparison to commercial BC hydrogel according to its potential for wound dressing applications.

### Low-Grammage Nanocellulose and BC Hydrogels for Wound Dressing Applications

**Figure 5:**
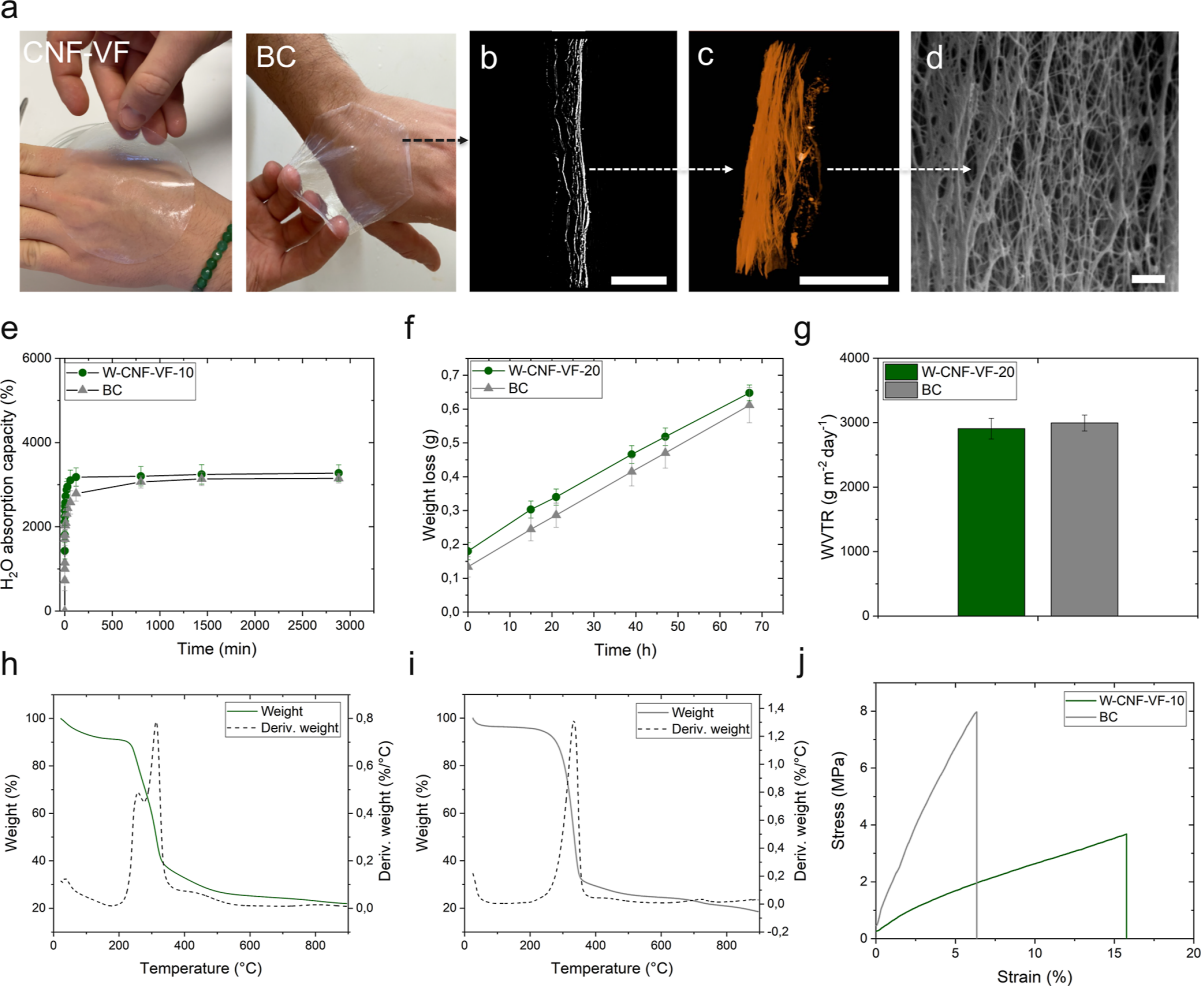
(a) Photographs of W-CNF-VF at a grammage of 10 g m^–2^ and BC hydrogels applied on hand. (b) XRT 2D reconstruction scale bar: 100 μm, (c) XRT 3D reconstruction scale bar: 100 μm and (d) SEM image scale bar: 1 μm of BC hydrogel cross section. W-CNF-VF and BC (e) absorption capacity in water, (f) weight loss, and (g) WVTR. TGA/derivative thermogravimetric curves for (h) W-CNF-VF and (i) BC samples. (j) Representative stress–strain curves.

From a wound dressing perspective, the W-CNF-VF hydrogel with the lowest grammage of 10 to 20 g m^–2^ were compared to commercial BC in terms of structure, absorption and retention capacity, thermal stability, and mechanical properties; the results are summarized in Figure 5.

The handling and transparency of the W-CNF-VF and BC hydrogels can be observed in Figure 5a. BC revealed a layered structure upon XRT 3D reconstruction (Figure 5b,c), also observed in Figure S5 (Supporting Information), similar to what was observed for the nanocellulose hydrogel (Figure 3b–d). However, by studying the BC structure at a higher magnification using SEM, a highly interconnected 3D network structure typical of BC was observed, which is shown in Figure 5d.

The liquid absorption capacities of the W-CNF-VF-10 and BC hydrogels were evaluated in water (Figure 5e) and in a PBS solution and PBS combined with a BSA solution and water (Figure S6, Supporting Information) to more closely resemble the physiological condition and wound environment. Overall, it can be observed from Figures 5e and S6, Supporting Information that the W-CNF-VF-10 hydrogel had an absorption capacity comparable to that of BC in the three media tested. Equilibrium absorption was reached within 1 h and the highest value was observed in water (∼3300%), followed by the BSA-PBS solution (∼1600%) and the PBS solution (∼1300%). The W-CNF-VF-10 and BC hydrogels also displayed a comparable ability to retain the water after absorption, as observed from Figure S7 (Supporting Information). Variables, such as temperature, pH, and the ionic strength of the surrounding medium, can affect the hydrogel swelling behavior.^70^ The W-CNF-VF and BC hydrogels are negatively charged as indicated by the negative ζ-potentials of −52 ± 2 for W-CNF and −18 ± 0 for BC. The effective charge value of BSA at a pH value higher than its isoelectric point (4.7) has been reported to be approximately −7.0 mV^71^ once the BSA solution is prepared in water. Thus, the ionic interactions among the charged moieties on the hydrogel surface, electrolytes in the PBS solution, and charged groups in the BSA molecules may have counteracted the swelling of the structure, thereby decreasing the liquid absorption capacity. This behavior is in agreement with the results of previous studies that reported a reduction in the liquid absorption capacity of ionic hydrogels in PBS and BSA solutions compared to that in distilled water.^31,72,73^

WVTR was measured by monitoring the weight loss over time to further evaluate the moisture maintenance, i.e., the ability to provide a moist wound environment (Figure 5f,g). From Figure 5g, the WVTRs were observed to be 2906 ± 160 and 2994 ± 124 g m^–2^ day^–1^ for W-CNF-VF-20 and BC hydrogels, respectively. Values above the range of 2000–2500 g m^–2^ day^–1^ provides adequate moisture transmission for optimal healing, without incurring in dehydration and scabbing (WVTR > 2500 g m^–2^ day^–1^), or in exudate retention and maceration (WVTR < 2000 g m^–2^ day^–1^).^74^

It is crucial for a successful wound dressing to absorb and retain wound exudates quickly and with an adequate capacity to reduce the risk of bacterial infection and promote the healing process.^75,76^ Therefore, the similar liquid absorption and retention behavior of the CNF-VF hydrogel with the BC hydrogel used here, which is already a commercial product, indicates the potential of the CNF-VF hydrogel for wound dressing applications.

The thermal stability of the hydrogels was evaluated using TGA (Figure 5h,i). An initial decrease in weight of approximately 10% was observed around 100 °C, owing to moisture loss for both W-CNF-VF and BC; thereafter, the materials exhibited, overall, stable behavior up to approximately 200 °C. This indicates that these materials have the required thermal stability for use as wound dressings in contact with the body or for steam sterilization.^76,77^

The tensile test showed that BC displayed approximately 140% higher strength and 75% higher elastic modulus than the W-CNF-VF hydrogel, which displayed 80% higher elongation to break (Figure 5j). The highly interconnected 3D network structure of BC (Figure 5d) is known to provide outstanding mechanical performance.^34^ Although BC assembles into a layered structure, its intrinsic network displays a more effective load transfer that contributes to its higher strength and stiffness than the W-CNF-VF hydrogel. This is also supported from the rheology assessment of W-CNF-VF-20 and BC hydrogels provided in Figures S8–S10 (Supporting Information), where BC displays a higher storage modulus, that corresponds to an increase in network strength compared to W-CNF-VF-20 upon cyclic compression Figure S10 (Supporting Information). Both materials displayed viscoelastic behavior with slower relaxation kinetics observed for W-CNF-VF-20 hydrogel compared to BC, but with similar relative axial force remaining.

The elastic modulus of skin varies considerably depending on the type and conditions of the mechanical test and for the tensile test, it ranges from approximately 5 to 20 MPa.^78^ The elastic modulus of the W-CNF-VF-10 hydrogel is within the upper limit of this range at 19.7 MPa, further signifying its potential for wound dressing applications.

**Figure 6:**
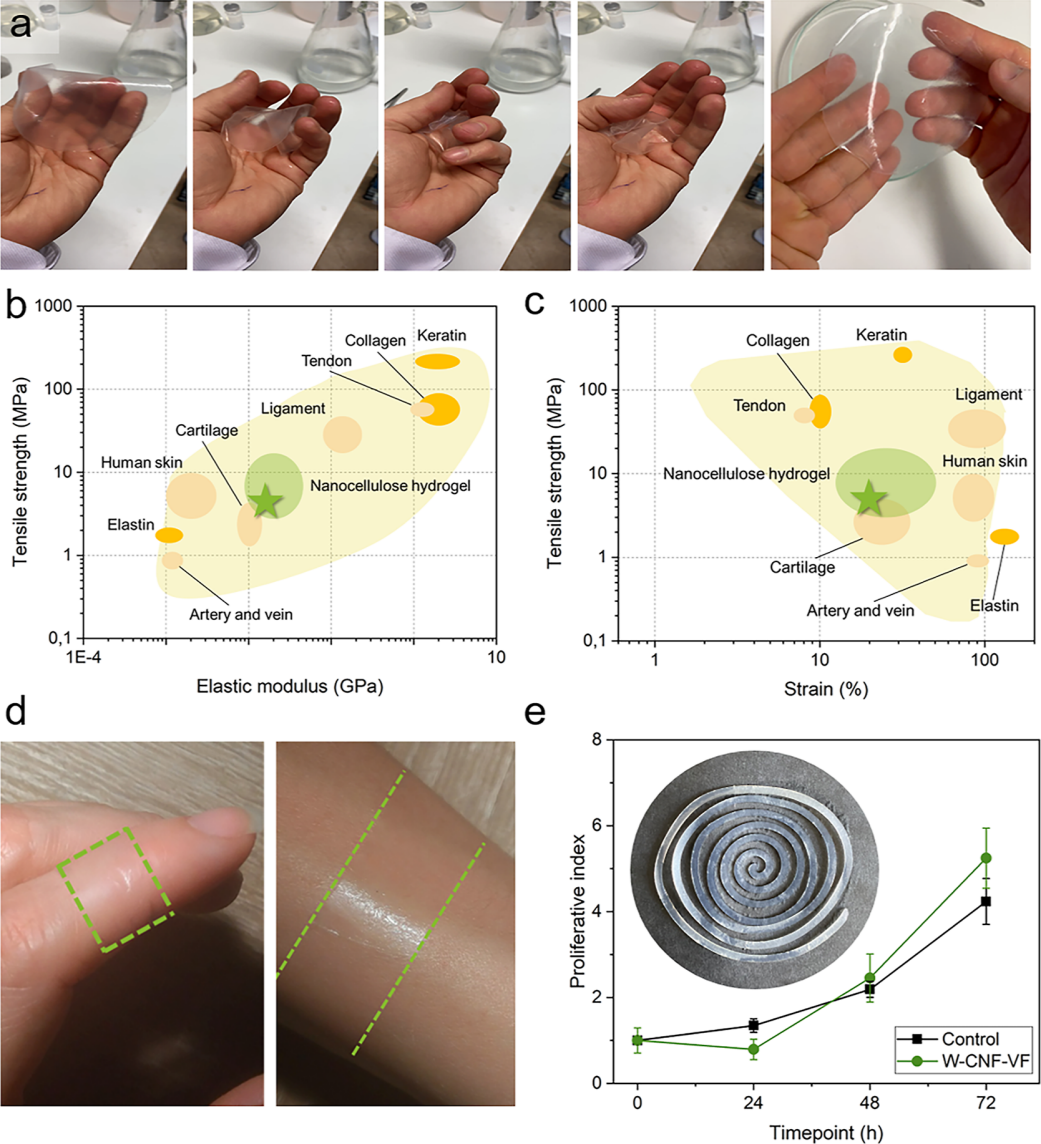
W-CNF-VF hydrogel after self-assembly. (a) Photographs upon handling, Ashby plot of (b) strength as a function of elastic modulus, and (c) strength as a function of strain compared to the soft tissue and its building blocks. (d) Photograph after application on finger and arm in the form of a hydrogel strip. (e) Effects of hydrogel on fibroblast proliferation (*n* = *5*), inset: laser-formed hydrogel into a spiral shape.

The potential use of self-assembled nanocellulose hydrogels for wound dressing applications was evaluated in a larger context by comparing the mechanical properties of soft tissue and its natural building blocks, together with an initial assessment of hydrogel cytocompatibility and general characteristics such as handling, forming, and applying the hydrogel, as presented in Figure 6.

As shown in Figure 6, the hydrogels were easily handled owing to their flexibility, shape retention (Figure 6a), conformability, and transparency (Figure 6d). Furthermore, the hydrogels could be easily formed into different shapes (Figure 6d,e inset). The tensile strength as a function of the elastic modulus (stiffness) and strain (elongation to break) of soft tissues and their structural building blocks are visualized in an Ashby plot together with nanocellulose hydrogels in Figure 6b,c, respectively. The green star represents the W-CNF-VF hydrogel at a grammage of 10 g m^–2^ and the range of mechanical properties for self-assembled nanocellulose hydrogels according to their varying grammage is visualized by the green bubble (Figure 6b,c). Overall, the nanocellulose hydrogel displayed a strength comparable to that of human skin; meanwhile, the elastic modulus was closer to that of cartilage (Figure 6b). Soft tissues that are predominantly built up from collagen, elastin, and proteoglycans (cartilage, ligament, skin, artery, and muscle) generally display lower tensile strengths but higher strain.^79^ The nanocellulose hydrogels displayed lower strain in comparison to the human skin; however, the ability to design hydrogels at various grammages can allow us to tune their ability to be elongated at ranges from approximately 15 to 45%.

An initial assessment of cytocompatibility was performed to evaluate the potential use of nanocellulose hydrogels for wound dressing applications. A proliferation assay in which viable cells were counted over a 72 h time period was employed for the analysis of the cell cultures (Figure 6e). An increasing number of fibroblasts was observed over time, as shown by the increased proliferative index for both nanocellulose and the control. The proliferation of fibroblasts was comparable to that of the control throughout the experiment, with a significantly higher proliferative index after 72 h (Figure 6e) of 5.25 ± 0.70 for nanocellulose, compared to 4.24 ± 0.53 for the control (*p* = 0.0019), indicating no cytotoxic side effects for the nanocellulose hydrogels. No significant differences were found at the other time points.

## Conclusions

The self-assembly of nanocellulose hydrogels from wood offers great potential for wound dressing applications. By applying vacuum-assisted filtration, the up-scalable and sustainable hydrogels excelled at mimicking commercial BC properties. The choice of raw material for nanocellulose separation and subsequent assembly into hydrogels was found to be an important factor that influenced the network formation ability between the nanofibers and the overall hydrogel structure stability. Self-assembly via vacuum-assisted filtration and suspension casting revealed a difference in the hydrogel structure and interconnection between the layers depending on the technique applied. The casted hydrogel exhibited a less interconnected layered structure upon swelling and, as a result, displayed an absorption capacity three times higher than that of the vacuum filtration technique. The hydrogel grammage also dictated the hydrogel properties, where both the mechanical properties and absorption capacity could be adjusted by changing the number of nanofibers that were allowed to self-assemble. The W-CNF-VF hydrogel was further tested in different solutions to more closely resemble the physiological condition and wound environment, and the liquid absorption capacity was found to be comparable to that of BC for all liquids. Also, the ability to maintain a moist environment by retaining water in the hydrogel structure was mimicking the behavior of the BC hydrogel. The thermal stabilities of the two materials were furthermore comparable and showed potential for use as wound dressings in contact with the body and up to steam sterilization temperatures. The mechanical properties of W-CNF-VF revealed a more flexible network with lower strength and stiffness than those of BC. In summary, the self-assembly of nanocellulose hydrogels demonstrates an up-scalable and sustainable approach that exploits the intrinsic characteristics of nanocellulose to design a completely bio-based hydrogel that is as strong as the human skin. Nanocellulose hydrogels combine attractive functions, such as design freedom, high absorption and reabsorption, flexibility, conformability, thermal stability, and transparency, while being nontoxic and mechanically stable in moist conditions resembling that of wound fluid, thereby being appropriate for wound dressing applications.

## Supporting Information Available

AFM height images along with size distribution and mean width of nanofibers obtained from wood and pulp, cyclic compression properties of W-CNF-VF hydrogel sample at 120 g m^–2^ in wet conditions, photographs of self-assembled W-CNF network obtained through suspension casting and vacuum-assisted filtration after air drying and in the hydrogel state (wet condition), absorption capacity of W-CNF-SC and W-CNF-VF hydrogels at 20 g m^–2^ after cyclic drying and re-swelling, thickness of the hydrogels in dry and wet states, 2D XRT reconstruction of BC hydrogel structure, absorption capacity as a function of time for W-CNF-VF-10 and BC in PBS solution and PBS-BSA solution, WRR as a function of time for W-CNF-VF-10 and BC hydrogels, axial force measurements as a function of time for VF-W-CNF-20 and BC hydrogels, axial force measurements as a function of step time and relative axial force remaining as a function of time for VF-W-CNF-20 and BC hydrogels, and storage and loss modulus as a function of time for VF-W-CNF-20 and BC hydrogels (PDF)
