# Supplementary material for: Self-Assembly of Nanocellulose Hydrogels Mimicking Bacterial Cellulose for Wound Dressing Applications
Source: Biomacromolecules. 2023 Apr 25;24(5):2264–77. doi: 10.1021/acs.biomac.3c00152 (PMC10170512; doi:10.1021/acs.biomac.3c00152)
Supplement: Supplementary file 1 — bm3c00152_si_001.pdf [file bm3c00152_si_001.pdf]

Supporting Information for

Self-Assembly of Nanocellulose Hydrogels Mimicking Bacterial Cellulose for Wound Dressing Applications

Linn Berglund[a], Paula Squinca[a,b], Yağmur Baş[a], Elisa Zattarin[c], Daniel Aili[c], Jonathan Rakar[d], Johan Junker[d], Annika Starkenberg[d] Mattia Diamanti[a], Petter Sivlér[e], Mårten Skog[e], Kristiina Oksman[a,f,g]*

[a] Department of Engineering Sciences and Mathematics, Luleå University of Technology, SE 97187, Luleå, Sweden

[b] Embrapa Instrumentation, Rua XV de Novembro 1452, 13561-206, São Carlos, SP, Brazil

[c] Department of Physics, Chemistry and Biology (IFM), Linköping University, Linköping, SE-581 83 Linköping, Sweden

[d] Center for Disaster Medicine and Traumatology, Department of Biomedical and Clinical Sciences, Linköping University, SE-581 85 Linköping, Sweden

[e] S2Medical AB, SE-58273 Linköping, Sweden

[f] Mechanical & Industrial Engineering, University of Toronto, 5 King's College Road, Toronto, ON M5S 3G8, Canada.

[g] Wallenberg Wood Science Center (WWSC): Luleå University of Technology, SE 97187 Luleå Sweden

*Corresponding author

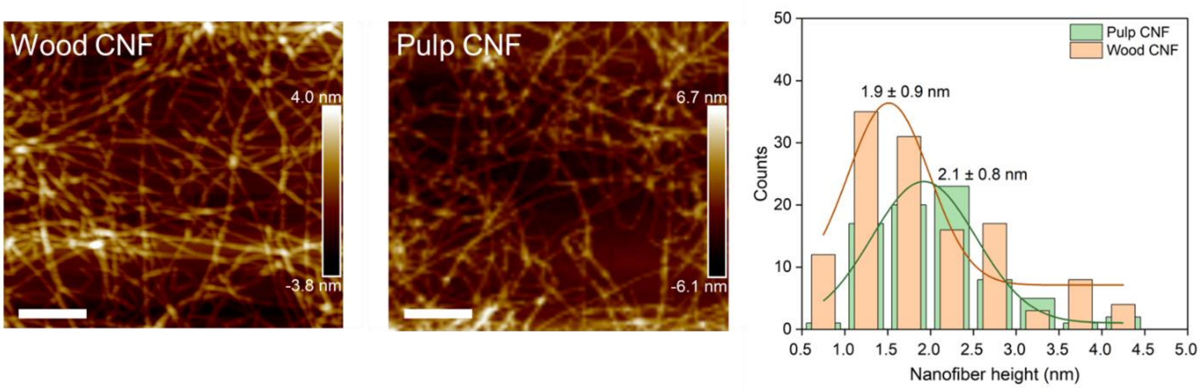

Figure S1. AFM height images and the measured size distribution (nanofiber width from height images) of W-CNF and P-CNF, respectively. Scale bar corresponds to 200 nm.

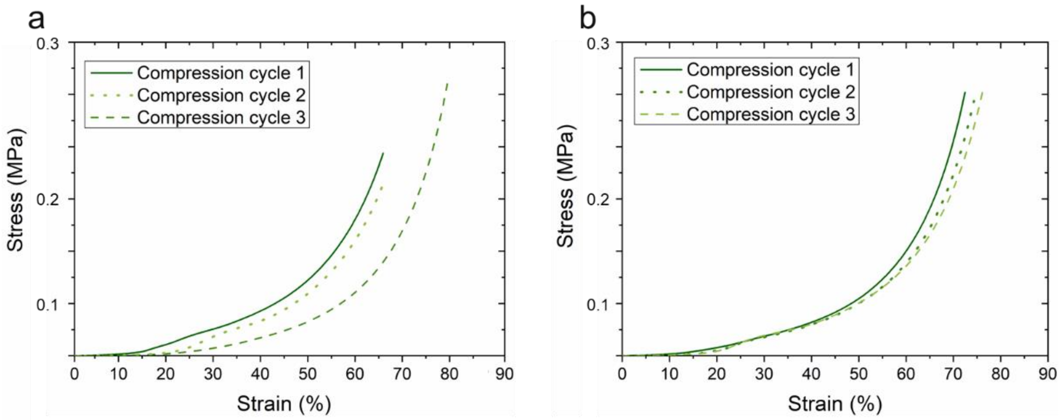

Figure S2. Cyclic compression test of the 120 g m^{-2} W-CNF-VF hydrogel sample for three compression cycles. a) Samples were left in air for a dwell time of 5 min between the compression cycles, b) the samples were immersed in water for 5 min between the cycles in order to study the hydrogel recovery.

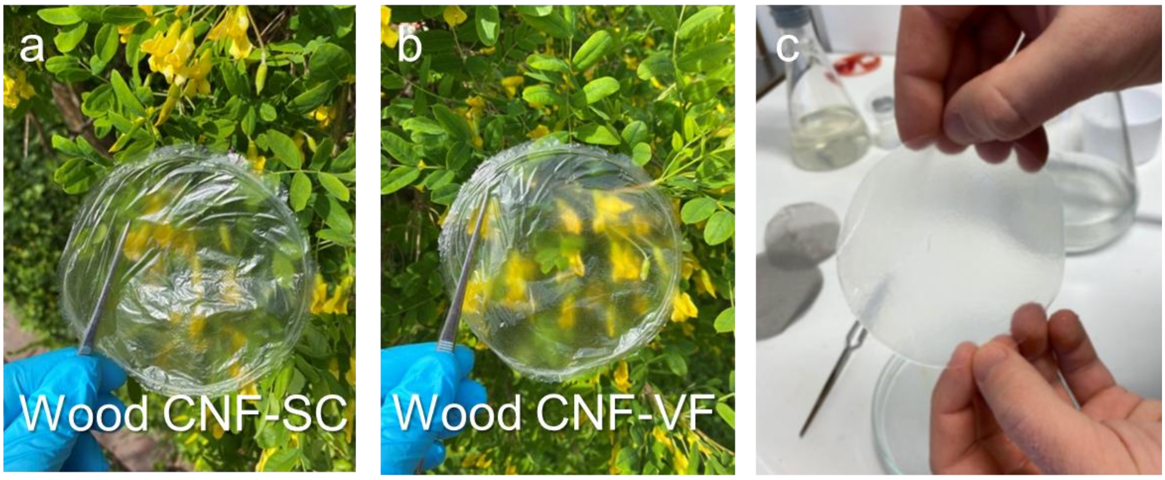

Figure S3. Photographs of self-assembled W-CNF network a) via solvent casting after air drying in RT, b) via vacuum assisted filtration after air drying in RT, and c) representative photograph of hydrogel state at grammage 10 g m^{-2}.

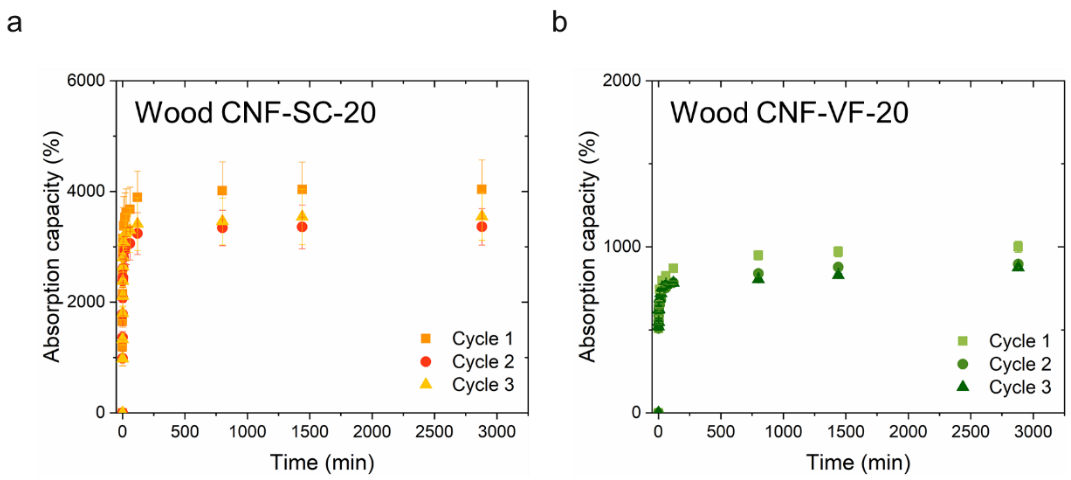

Figure S4. Absorption capacity after cyclic drying and re-swelling of the hydrogels at grammage 20 g m^{-2} a) W-CNF-SC and b) W-CNF-VF.

Table S1. Thickness of the hydrogels at dry and wet state (equilibrium swelling capacity) for grammage 10 and 20 g m^{-2} obtained by suspension casting and vacuum assisted filtration.

	Thickness dry (µm)	Thickness wet (µm)
W-CNF-SC-10	13.2 ± 0.8	974.1 ± 56.4
W-CNF-SC-20	17.5 ± 2.6	603.8 ± 69.0
W-CNF-VF-10	17.3 ± 4.9	152.8 ± 12.6
W-CNF-VF-20	27.4 ± 7.4	136.1 ± 6.9

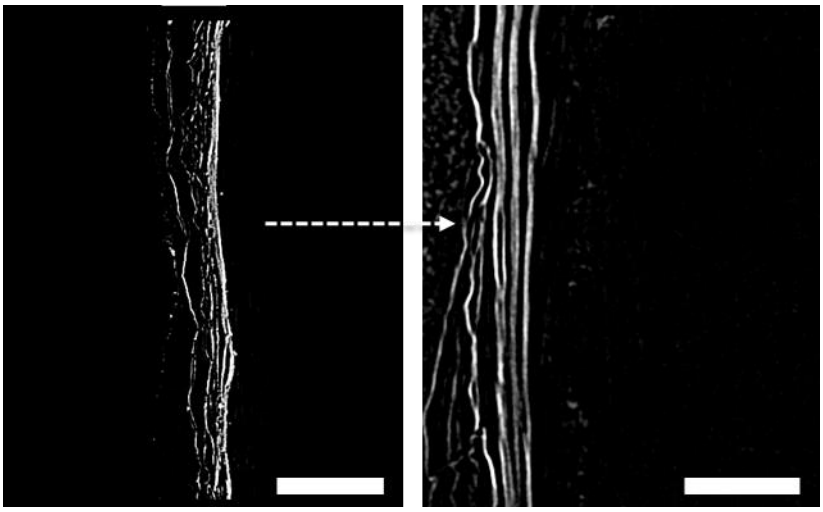

Figure S5. 2D XRT reconstruction of BC. Scale bar: 100 µm (to the left). Scale bar: 50 µm (to the right).

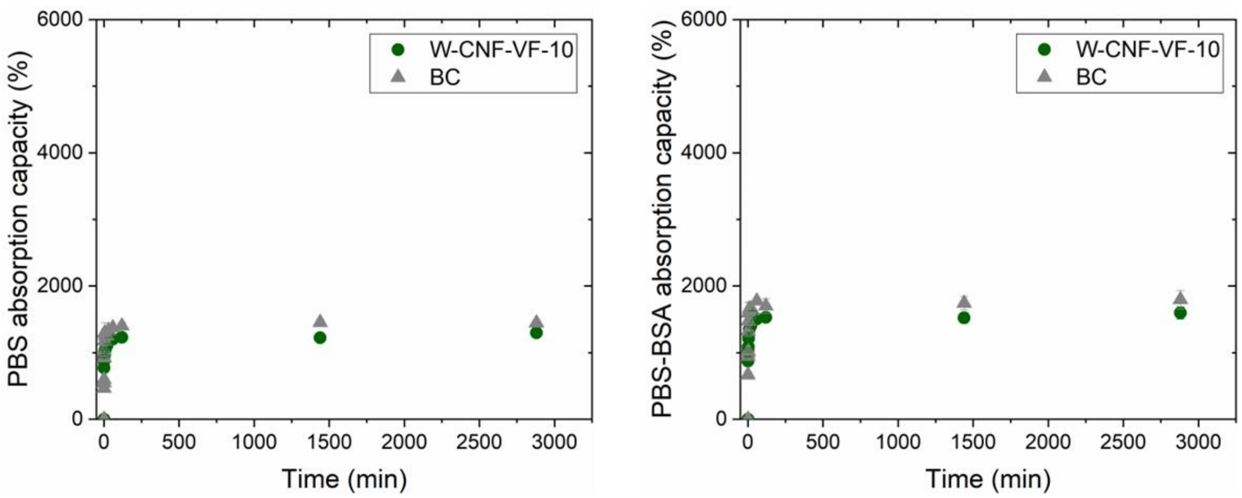

Figure S6. W-CNF-VF-10 and BC absorption capacity as a function of time in PBS solution, and PBS-BSA solution, respectively.

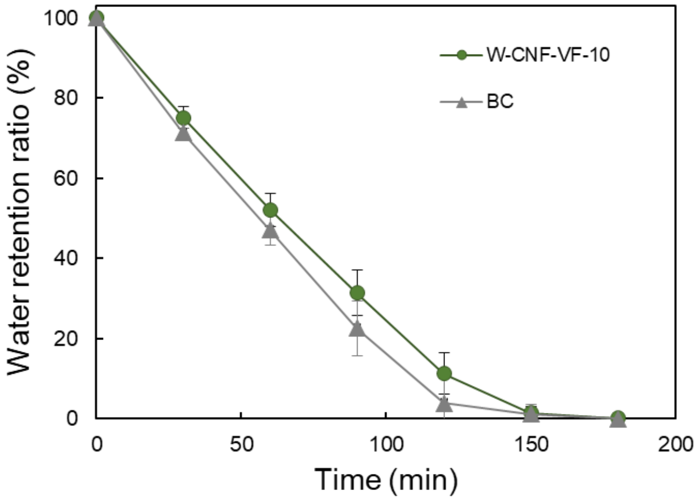

Figure S7. Water retention ratio as a function of time for W-CNF-VF-10 and BC hydrogel, respectively.

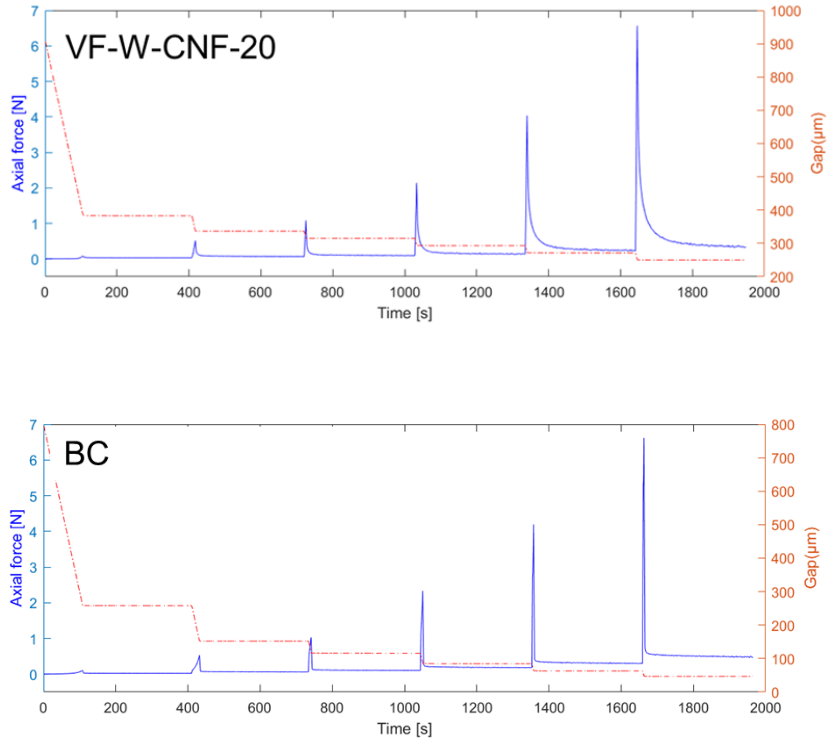

Figure S8. Axial force measurements as a function of time for W-CNF-VF-20 and BC.

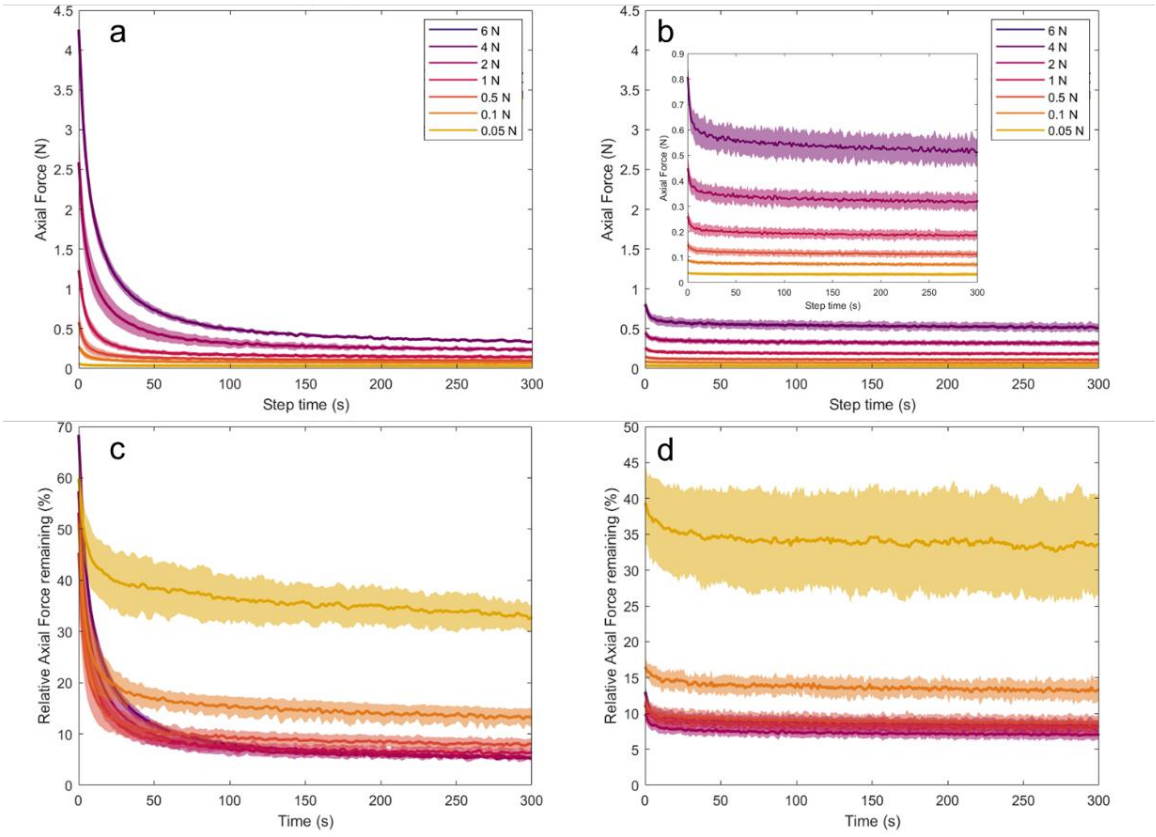

Figure S9. Axial force measurements as a function of step time for a) W-CNF-VF-20and b) BC and relative axial force remaining as a function of time for c) W-CNF-VF-20and d) BC.

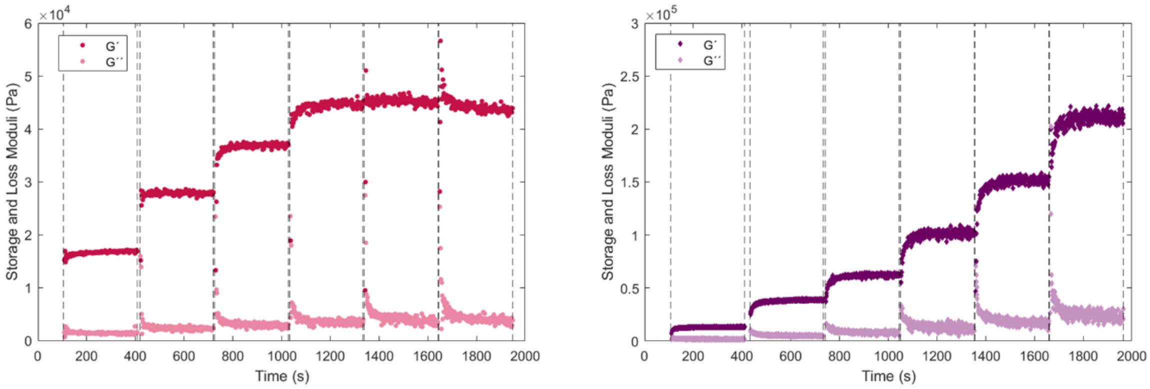

Figure S10. Storage and loss modulus as a function of time for W-CNF-VF-20 and BC, respectively.
